# Evolution and Detection of β-Lactamases in *Helicobacter pylori* Isolates from High- and Low-Risk Southwestern Colombian Areas for Gastric Cancer

**DOI:** 10.3390/antibiotics15100943

**Published:** 2026-09-23

**Authors:** Kevin Andres Guzman, Jose Dario Portillo Miño, Sandra Cifuentes, Arnoldo Riquelme, Alvaro Pazos

**Affiliations:** 1 Grupo de Investigación Salud Pública, Centro de Estudios en Salud, Universidad de Nariño, Pasto 520002, Colombia; alpazmo@yahoo.com; 2 Grupo de Investigación en Ciencia de la Salud (GICIENSA), Facultad de Salud, Programa de Medicina, Universidad de Nariño, Pasto 520002, Colombia; josedariopm33@gmail.com; 3 Grupo de Investigación en Enfermedades Infecciosas y Cáncer (GINFYCA), Centro de Investigaciones Clínicas, Fundación Hospital San Pedro, Pasto 520002, Colombia; sandrapcifuentesa@gmail.com; 4 Departamento de Gastroenterología, Facultad de Medicina, Pontificia Universidad Católica de Chile, Santiago 8320000, Chile; a.riquelme.perez@gmail.com; 5 Departamento de Biología, Facultad de Ciencias Exactas y Naturales, Universidad de Nariño, Pasto 520001, Colombia

**Keywords:** *Helicobacter pylori*, β-lactamases, *blaTEM*, *blaSHV*, amoxicillin resistance, Colombia

## Abstract

Gastric cancer (GC) is one of the leading causes of cancer-related mortality worldwide, with *Helicobacter pylori* infection recognized as its main risk factor. Eradication therapy is the cornerstone of GC prevention; however, increasing antimicrobial resistance (AMR) threatens the effectiveness of amoxicillin-based regimens. This study investigated the occurrence and evolutionary distribution of β-lactamase genes in Colombian *H. pylori* isolates. A total of 67 *H. pylori* isolates from Colombian regions with high and low GC risk were analyzed. The presence of *blaTEM* and *blaSHV* genes was assessed using PCR, following in silico primer validation against complete *H. pylori* genomes. Phylogenomic relationships were reconstructed using the Neighbor-Joining method in MEGA XI, with the best-fitting evolutionary model selected according to (AIC). The GTR + G + I model was identified as the optimal model and was subsequently used for phylogenetic reconstruction. The resulting tree was visualized using iTOL. This study reports, for the first time in Colombia, that the presence of β-lactamase genes in *H. pylori*. *blaTEM* and *blaSHV* were detected in 9 of 48 Andean isolates (18.75%), whereas no β-lactamase genes were identified in isolates from the low-risk Pacific region. These genes were associated with genetic potential for amoxicillin resistance. Phylogenomic analysis identified a distinct Andean lineage in which all β-lactamase-positive isolates clustered. β-Lactamase genes in Colombian *H. pylori* are geographically restricted to high-risk Andean populations and associated with a defined phylogenetic lineage. These findings provide new insights into the evolution of amoxicillin resistance and support the implementation of genomic surveillance to optimize eradication strategies and improve precision management of *H. pylori* infection.

## 1. Introduction

Gastric cancer (GC) is the fifth most common malignant neoplasia and the fourth leading cause of cancer and death globally. Also globally, the incidence and mortality rates and GC risk are distinct [1,2]. GC is the leading cause of mortality in Colombia and shows a clear geographical distribution in terms of incidence. It is higher in mountainous regions and lower in the coastal areas of the country [3,4,5].

*Helicobacter pylori* (*H. pylori*) infects 70–90% of individuals in developing countries and 25–50% in developed countries, with prevalence influenced by socioeconomic and environmental factors. Although most infected individuals develop chronic gastritis, a subset progresses to peptic ulcer disease, MALT lymphoma, or GC [6]. *H. pylori* infection is the main trigger factor involved in the development of GC [4,5,7]. Other risk factors involved in the complex interaction include environmental and hygiene-sanitary factors; diet; host immune response; *H. pylori* coevolution; microbiota disruption; and human genetic susceptibility, leading to a maladaptive human response of gastric pathophysiology [8,9,10,11,12,13].

The state of Nariño in southwestern Colombia is considered the example of the “Colombian enigma.” This concept emerged to describe two areas with equal prevalence of *H. pylori* (90%) infection but with different GC incidences. In this context, we studied an Andean zone of high mountains inhabited by a population of Amerindian origin (67%) and a high GC incidence, which is considered one of the highest in the world (150/100.000 inhabitants), and a coastal zone with a population of mostly African origin (58%) with a low GC incidence (6/100.000 inhabitants) [11,14,15,16]. This discrepancy between these two areas is relevant to *H. pylori*-leading carcinogenesis. Therefore, elucidating the GC etiopathogenesis requires considering these unique geographical, sociocultural, and ethnic variations.

Amoxicillin remains a key component of first-line therapy due to its favorable pharmacodynamic profile; however, resistance is increasingly reported. *H. pylori* acquires amoxicillin resistance through altered penicillin-binding proteins, efflux pump overexpression, or β-lactamase production [17]. The *blaTEM* gene, in particular, is widely disseminated among pathogenic bacteria worldwide [18]. According to the recent report of the global Antibiotic Resistance (AMR) profile, Schultz et al. [19] showed that amoxicillin remains an exception, with resistance rates under 2% in 14 of 31 countries, although some countries have reported amoxicillin resistance rates of over 90%. In Colombia, the amoxicillin resistance is very heterogeneous according to each region, covering between 3.8% (Bogota) and 25.9% (Bogota and Nariño) [20].

*H. pylori* acquires resistance to β-lactams by modifying penicillin-binding proteins (PBPs), overexpressing efflux pumps, and, less frequently, producing β-lactamases encoded by genes such as *blaTEM* and *blaSHV*, which hydrolyze the β-lactam ring [21]. Previous studies in Antioquia, Colombia, did not detect these genes despite molecular evidence from biopsies [18,22]. In this context, we study the presence, evolution, and phylogenetic distribution of β-lactamase genes (*blaTEM* and *blaSHV*) in Colombian *H. pylori* isolates from regions with different GC risks to assess the amoxicillin resistance patterns.

## 2. Results

The circular phylogenetic tree illustrates the genetic diversity of *H. pylori* across different regions of the world, with color-coded groupings representing ancestral and derived lineages. African populations (hpAfrica2 and hspWAfrica) occupy the oldest branches, reflecting their greater genetic diversity and their role as an ancestral reservoir. In contrast, Eurasian (hspEAsia and hpEurope) and Native American (hspAmerindian) populations exhibit patterns of differentiation associated with human migrations and local adaptation. In this study, phylogenetic analysis of *H. pylori* revealed a clear differentiation between ancestral and derived lineages, with clusters defined according to geographic origin. A new subpopulation, hspColombia_PacificCoast, was identified among isolates from Pacific Coast of Colombia, whereas the hspColombia_Andes subpopulation was identified in isolates from the Andean region. Interestingly, all resistant isolates carried both the blaTEM and blaSHV genes. Phylogenetic analysis showed that two resistant isolates from the Andean region clustered with the hpEurope lineage, whereas the remaining resistant isolates belonged to the hspColombia_Andes subpopulation (Figure 1).

This study reports, for the first time in Colombia, the detection of β-lactamase genes in *H. pylori*. blaTEM and blaSHV were identified in 9 of 48 Andean isolates (18.75%), predominantly from regions at high GC risk, whereas no β-lactamase genes were detected in isolates from the low-risk Pacific region. ResFinder analysis predicted that these genes confer resistance to several β-lactam antibiotics, including ampicillin, cephalothin, piperacillin, and ticarcillin.

Three-dimensional structural analysis reveals the conformation of the protein under study through a ribbon diagram model, where α-helices connecting loops are clearly distinguishable. The spatial organization shows a compact architecture with differentiated domains, suggesting a specific functional distribution between N-terminal and C-terminal regions. At the center of the structure, a small ligand, represented as a rod model, is observed binding to key residues of the active site. The ligand’s proximity to the α-helices indicates a highly conserved binding environment, reinforcing the hypothesis that this protein plays an essential role in catalytic or regulatory processes with amoxicillin. Furthermore, regions of flexibility are identified in the connecting loops, which could facilitate dynamic conformational changes during interaction with substrates or inhibitors. This structural plasticity is a characteristic feature of proteins with enzymatic or regulatory activity.

Molecular docking analysis showed that the most favorable binding pose of amoxicillin with the BlaTEM enzyme corresponded to Cluster 0 (Rank 0), with a predicted binding free energy (ΔG) of −8.28 kcal/mol, indicating a stable ligand–protein interaction. The second-best binding pose (Cluster 1, Rank 0) exhibited a comparable binding affinity (ΔG = −8.02 kcal/mol), suggesting the presence of alternative stable conformations. Overall, the docking results support a strong interaction between amoxicillin and the BlaTEM active site (Figure 2).

Molecular docking analysis showed that amoxicillin bound favorably to both β-lactamases. The most stable binding pose for BlaTEM corresponded to Cluster 0 (Rank 0) with a predicted binding free energy (ΔG) of −8.28 kcal/mol, whereas the best pose for BlaSHV was identified in Cluster 1 (Rank 0) with a ΔG of −7.73 kcal/mol. The slightly lower ΔG observed for BlaTEM suggests a marginally stronger predicted binding affinity for amoxicillin compared with BlaSHV (Figure 3).

## 3. Discussion

This study, for the first time in Colombia, examines the presence, changes, and spread of *blaTEM* and *blaSHV* in samples from areas at high risk (Túquerres, Pasto, and La Florida) and low risk (Tumaco and Barbacoas) for gastric cancer. It uses in silico PCR, molecular docking, and phylogenomic analysis. Clinical trials of chemoprevention have significantly demonstrated that *H. pylori* eradication has a favorable impact on GC mortality [23,24,25]. The Colombian cohort study conducted by Piazuelo et al. [23], which revealed interesting findings after 20 years of follow-up, showed that GCA can revert to a non-atrophic state after at least six years of *H. pylori* eradication; however, GIM is usually irreversible in less advanced stages, except in patients with limited or complete GIM [23,26]. This is important due to the fundamental role of *H. pylori* infection in critically interrupting the progression of GPMC.

According to the Maastricht VI/Florence Consensus guidelines, standard bismuth-based quadruple therapy, comprising two antibiotics and an acid suppressant administered for 14 days, is the first-line treatment for *H. pylori* eradication and is adjusted depending on the local antibiotic resistance profile [27]. However, recently, the increase in global rates of resistance to amoxicillin has significantly reduced the effectiveness of this regimen [28]. A report issued in 2022 by Murray et al. [28] indicates that globally, AMR to amoxicillin has increased substantially to around 45%. In this context, the Maastricht VI/Florence agreement makes it clear that if antibiotic resistance is adjusted according to local AMR patterns and guidelines, it is necessary [27]. Therefore, there is an urgent need for alternative treatment regimens to combat the growing resistance to antibiotics, particularly to amoxicillin. In contrast, systematic and meta-analysis data on amoxicillin resistance across some WHO regions suggest that resistance estimates may reach 5% or more in other areas [29]. This finding is remarkable because the divergent distribution of heterogeneity is a prerequisite for global surveillance in each region and country.

The mechanism of action of amoxicillin weakens the bacterial cell wall through PBPs, which are crucial enzymes in bacterial cell wall synthesis, because it inhibits PBPs, preventing the cross-linking of peptidoglycan chains, leading to a weakened and unstable cell wall, allowing metronidazole to generate free radicals that damage DNA and clarithromycin to act directly on the inhibition of bacterial protein synthesis through its binding to the *50S* ribosomal subunit, blocking tRNA translocation during peptide chain elongation [20,30].

This finding makes it a particularly relevant example of how evolution and medical challenges interact. The discovery of *blaTEM* and *blaSHV* genes in different strains highlights that amoxicillin resistance is a widespread issue, affecting both worldwide treatment methods and medical practice in Colombia. The presence of β-lactam resistance genes (*blaTEM* and *blaSHV*), indicated in the tree using markers, reveals a distribution pattern not restricted to a single lineage but rather dispersed across several branches (hspColombia_Colombia and hpEurope). This suggests that resistance acquisition has occurred convergently, likely driven by selective pressure from the widespread use of amoxicillin and other β-lactams in different geographic contexts. In Colombian subpopulations, the detection of these genes indicates that resistance has also been incorporated into local lineages, which has direct implications for therapeutic efficacy in the region. These findings illustrate how the evolutionary history of *H. pylori* intertwines with the contemporary dynamics of antimicrobial resistance. Phylogenetic diversity reflects coevolution with human populations, while the spread of *blaSHV* and *blaTEM* genes demonstrates the pathogen’s ability to adapt to global clinical pressure. The identification of β-lactamase genes in Colombian lineages has direct implications for the efficacy of amoxicillin as part of standard therapy, which is compromised, reinforcing the need for local surveillance and for adapting treatment guidelines to the local epidemiology of Nariño (Colombia) and the urgency of alternative treatment strategies and antimicrobial stewardship programs [31]. In Latin America, the *H. pylori* screen-and-treat strategy had been implemented as a pilot program across countries, insofar as AMR surveillance is necessary and rigorous for guiding effective therapies according to experts’ group recommendations [31,32,33].

The docking results indicate that amoxicillin can interact favorably with both BlaTEM and BlaSHV proteins. BlaTEM exhibited the lowest predicted binding free energy (−8.28 kcal/mol), suggesting a slightly stronger interaction than BlaSHV (−7.73 kcal/mol). These findings are consistent with the ability of class A β-lactamases to recognize β-lactam antibiotics and support the potential contribution of these enzymes to amoxicillin resistance in *H. pylori*. This pattern suggests that the protein may participate in molecular recognition processes and regulation, whose activity depends on the conformational stability of adjacent domains, in accordance with previous studies of molecular dynamics that have demonstrated the importance of conformational flexibility in enzyme function [34]. The identification of a ligand in the active site supports the functional relevance of this region and opens the possibility of designing targeted inhibitors that block the interaction. Furthermore, the presence of flexible regions suggests a dynamic opening and closing mechanism, characteristic of proteins that require adaptability for their biological function.

In the context of *H. pylori* infection, the presence of β-lactam resistance genes (*blaTEM* and *blaSHV*) in different lineages reinforces the need for alternative therapeutic strategies. Global evidence indicates that amoxicillin resistance can reach up to 50% in some regions, significantly reducing the efficacy of standard regimens [28]. These findings, along with the structural characterization of key proteins, emphasize the necessity of integrating phylogenetic, structural, and clinical data for the design of new therapies. This demonstrates the need for personalized treatments that consider resistance and the host–pathogen interaction. Further studies are required to evaluate safety, optimal dosage, and long-term outcomes, particularly in resistant groups, as clinical validation is still lacking [35]. Furthermore, an additional challenge for primary prevention is vaccine development, which remains a challenging but promising field [36]. Preclinical models have shown that several vaccines in development can induce immunity, such as those using live attenuated vectors, DNA, and recombinant subunits. However, inconsistent protection and a weak mucosal immune response have hampered human trials. Given this scenario, *H. pylori* eradication remains the first option for primary strategies.

Although mutations in *pbp1* and other alterations affecting penicillin-binding proteins, cell wall biosynthesis, and membrane permeability are well-established mechanisms contributing to amoxicillin resistance in *H. pylori* [20], the detection of *blaTEM* and *blaSHV* in the present study suggests a potentially unexplored mechanism that warrants further investigation. Importantly, the identification of these genes should not be interpreted as evidence that β-lactamase production is a primary or established mechanism of amoxicillin resistance in *H. pylori*. Rather, their presence represents a novel genomic finding that raises the possibility of an additional resistance mechanism. Future studies should therefore investigate the contribution of *blaTEM* and *blaSHV* alongside established determinants such as *pbp1* mutations and other resistance-associated mechanisms. Functional and in vitro studies will be particularly important to determine whether these genes are expressed and whether their presence contributes directly to reduced amoxicillin susceptibility.

## 4. Materials and Methods

### 4.1. Obtaining and Bioinformatic Analysis of Helicobacter Pylori Isolates

This study was designed as a retrospective bioinformatic analysis of publicly available *H. pylori* whole-genome sequences. A total of 67 complete *H. pylori* genomes from Colombian isolates were retrieved from the PubMLST database (https://pubmlst.org/), where the genomes had been generated and deposited as part of previous sequencing projects. The available metadata were used to classify the isolates according to their geographic origin and the reported gastric cancer (GC) risk of the corresponding region. Forty-eight genomes originated from high-GC-risk Andean regions (Túquerres, Pasto, and La Florida), whereas 19 originated from low-GC-risk Pacific regions (Tumaco and Barbacoas).

The genomic sequences were analyzed to identify the presence of the *blaTEM* and *blaSHV* β-lactamase genes. Candidate sequences were identified through BLAST (Bethesda, MD, USA) searches against the National Center for Biotechnology Information (NCBI) database using the corresponding reference sequences for *blaTEM* (NG_050145, EU726527.1) and *blaSHV* (MG653180.1). The analysis was performed in silico using the complete genomes deposited in PubMLST; therefore, no conventional PCR amplification was performed as part of the present study. Candidate gene sequences were considered identified when high-confidence sequence similarity (>95%) and complete or near-complete alignment with the reference genes were observed. The nucleotide sequences identified in the Colombian *Helicobacter pylori* genomes were subsequently extracted and analyzed to characterize their distribution among the available geographic groups, utilizing specific primers for the identification of the $\beta$-lactamase genes (*blaTEM* and *blaSHV*) (Table 1).

An analysis was also performed using ResFinder as a predictor of *H. pylori* resistance to other potential antibiotics that might not work given the presence of these *blaTEM* and *blaSHV* genes with the online tool Global Pathogen Analysis Platform (https://genepi.food.dtu.dk/).

### 4.2. Phylogenetic Analysis of Helicobacter Pylori Based on Whole Genome

All complete genomes were imported into the BIGSdb database [37] for gene alignment, using the CDS sequences of *H. pylori* strain 26695 as a reference. The genomic comparator array was exported for phylogenetic reconstruction using the Neighbor-Joining method in MEGA XI. The best-fitting evolutionary model was selected based on the Akaike Information Criterion (AIC), which identified the GTR + G + I model as the most appropriate for the dataset.

### 4.3. Molecular Docking of Helicobacter Pylori β-Lactamases and Amoxicillin

Amoxicillin, downloaded and cleaned from the Protein Data Bank (PDB ID: 6I1F) (https://www.rcsb.org/), was used as the ligand. The *blaTEM* and *blaSHV* genes were identified in *H. pylori* isolates from Nariño, Colombia, by PCR and confirmed by BLAST analysis. Consensus nucleotide sequences were generated for each gene based on the sequences obtained from the identified *blaTEM* and *blaSHV* variants. The corresponding consensus protein sequences were subsequently used to predict the three-dimensional structures of the TEM and SHV β-lactamases using AlphaFold (https://alphafold.ebi.ac.uk/). Thus, the protein structures used as receptors were derived from consensus sequences and did not correspond to a specific clinical isolate or reference strain. Molecular docking was performed using the SwissDock online platform with default parameters, and the most suitable docking model was selected based on the lowest Gibbs free energy (http://old.swissdock.ch/docking) (Accessed on 21 February 2026).

## 5. Conclusions

This study provides the first evidence of the *blaTEM* and *blaSHV* β-lactamase genes in Colombian *Helicobacter pylori* isolates and demonstrates their restriction to high-gastric-cancer-risk Andean populations. These findings improve our understanding of the evolution of amoxicillin resistance (18.75%) in *H. pylori* and highlight the importance of integrating genomic surveillance into antimicrobial resistance monitoring to support evidence-based eradication strategies in high-risk regions.

## Figures and Tables

**Figure 1:**
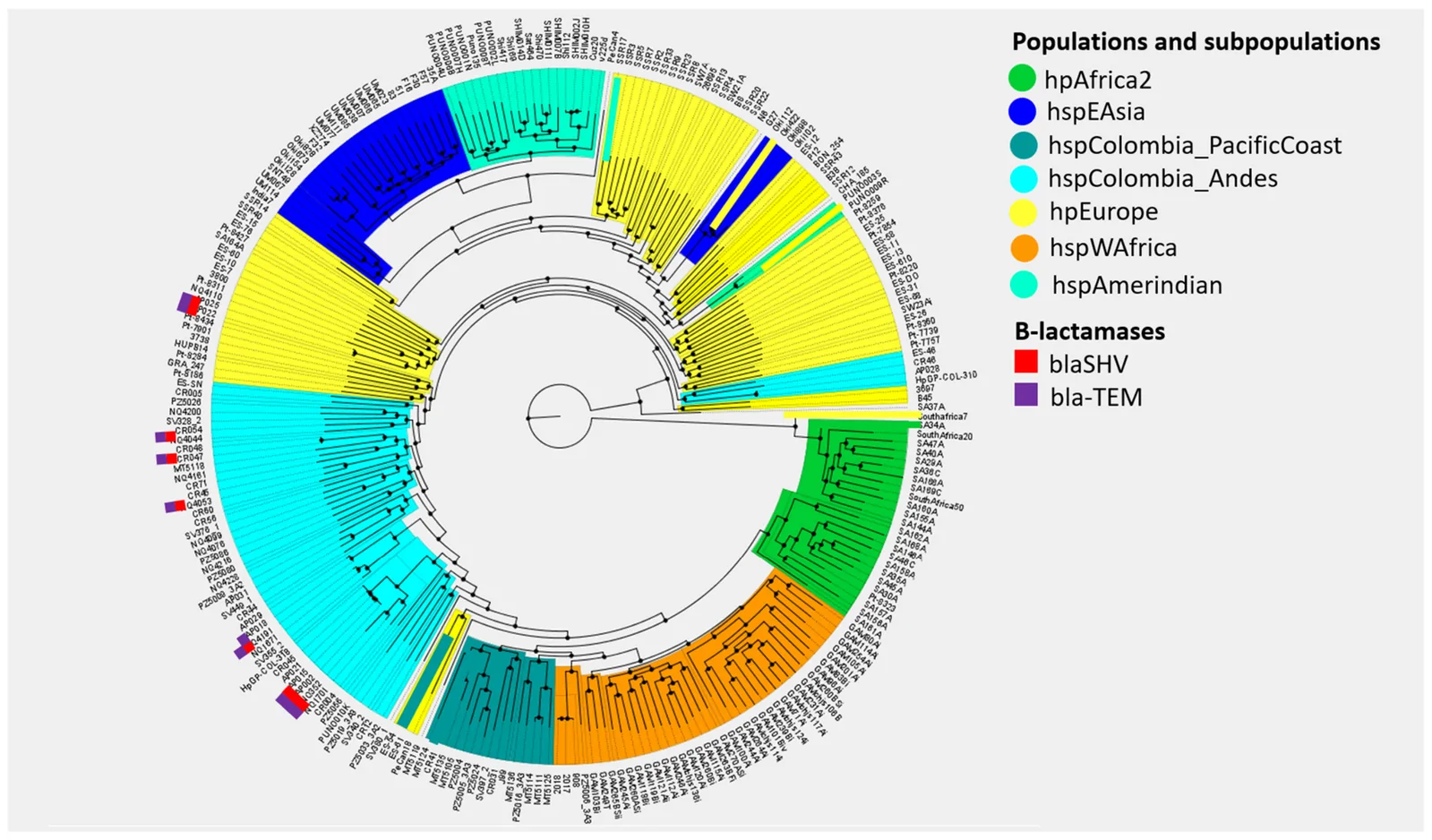
Phylogenomic tree of *Helicobacter pylori* isolates from populations with high and low risk of gastric cancer in Colombia.

**Figure 2:**
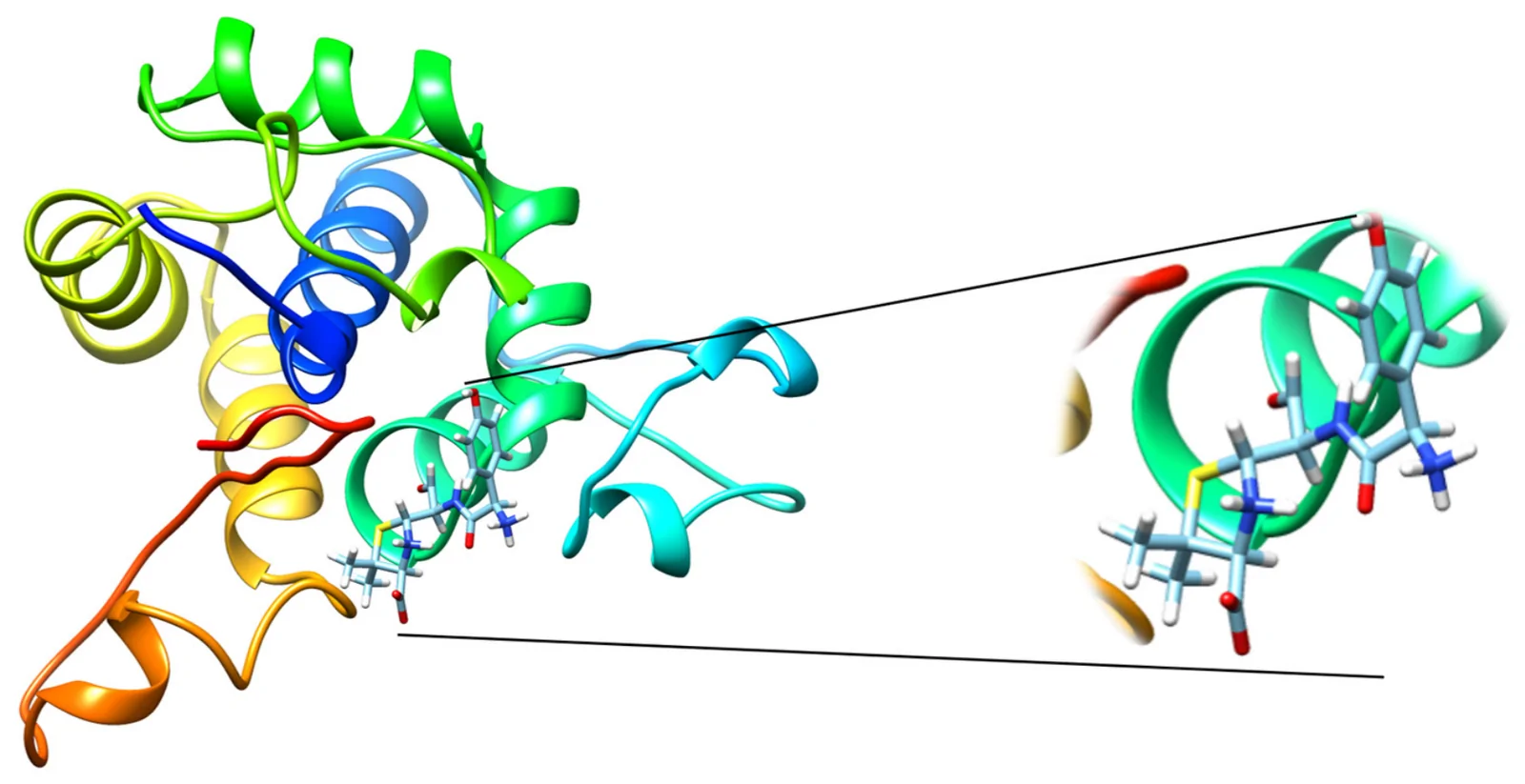
Structural dimension of the protein under study the BlaTEM enzyme isolated from *Helicobacter pylori* in Nariño, Colombia complexed with the ligand amoxicillin, visualized through a ribbon diagram model.

**Figure 3:**
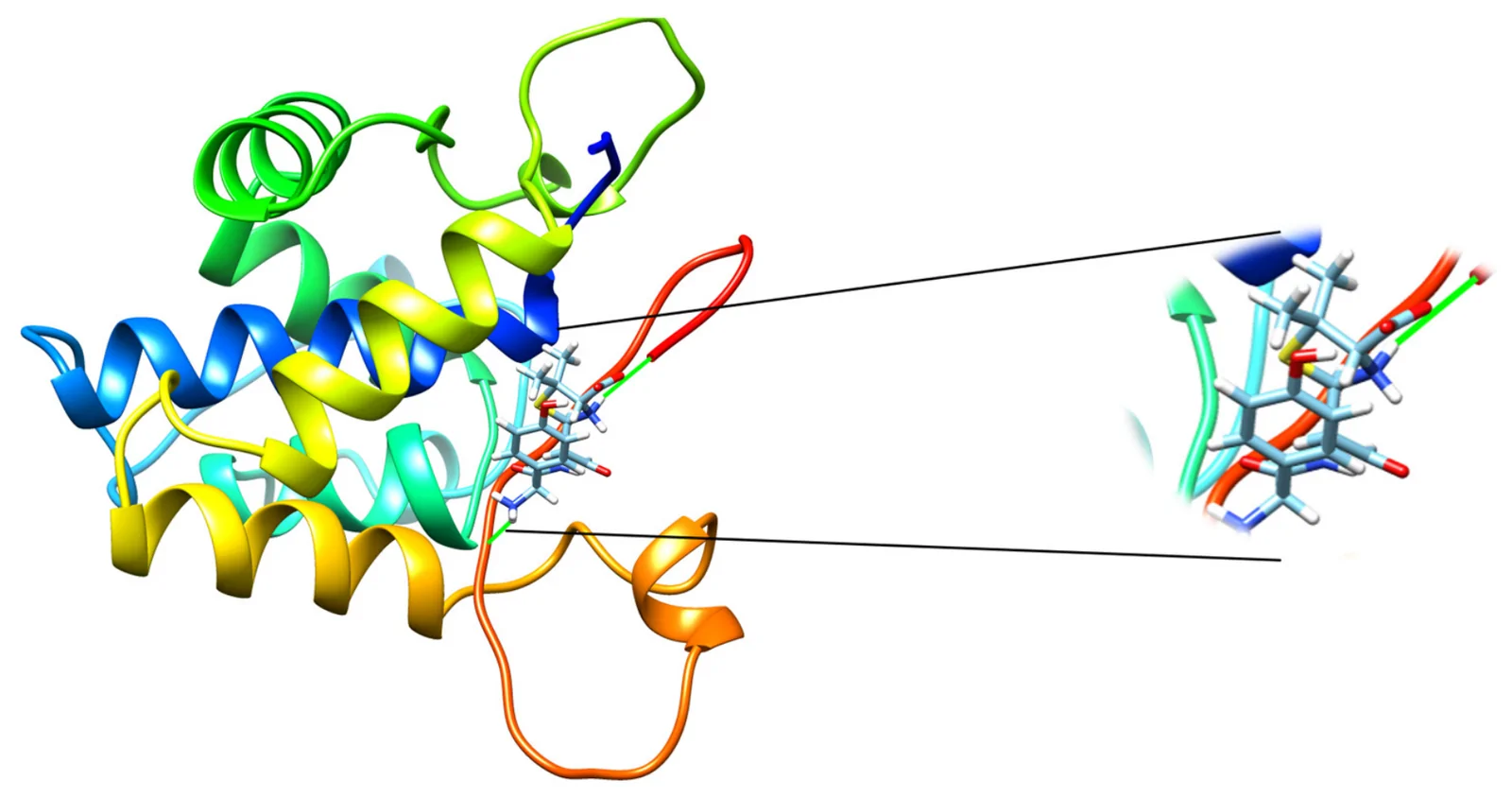
Structural dimension of the protein under study the BlaSHV enzyme isolated from *Helicobacter pylori* in Nariño, Colombia, complexed with the ligand amoxicillin, visualized through a ribbon diagram model.

**Table 1:** Primers used for the identification of *bla*-TEM and *bla*-SHV genes of *Helicobacter pylori* from Colombia.

β-Lactamases	Primers
***blaTEM***	ATGGGG-GATCATGTAACTCG
	GATACGG-GAGGGCTTACCAT
***blaSHV***	TTCGTGTCGCCCTTATTCCC
	CCCAACTGATCTTCAGCATCT

## Data Availability

Data Availability are available in: https://pubmlst.org/.
